# Editorial: Hypoxia as a therapeutic tool in search of healthy aging

**DOI:** 10.3389/fphys.2022.1112129

**Published:** 2023-01-12

**Authors:** Alba Camacho-Cardenosa, Johannes Burtscher, Martin Burtscher, Marta Camacho-Cardenosa

**Affiliations:** ^1^ PROFITH (PROmoting FITness and Health through Physical Activity) Research Group, Sport and Health University Research Institute (iMUDS), Department of Physical Education and Sport, Faculty of Sport Sciences, University of Granada, Granada, Spain; ^2^ Institute of Sport Sciences, University of Lausanne, Lausanne, Switzerland; ^3^ Institute of Sport Science, University of Innsbruck, Innsbruck, Austria; ^4^ Clinical Management Unit of Endocrinology and Nutrition - GC17, Maimónides Biomedical Research Institute of Cordoba (IMIBIC), Reina Sofía University Hospital, Córdoba, Spain

**Keywords:** hypoxia, oxygen homeostasis, aging, exercise, frail elderly

## 1 Hypoxia-Based interventions and ageing: Benefits comparable and complementary to exercise

Physical activity is considered one of the most powerful life-style interventions to promote healthy ageing (Sallis, 2015). However, older adults rarely meet the recommended levels of physical activity (Troiano et al., 2008; Izquierdo et al., 2021), often due to physical inability. With increasing life expectancies and growing prevalence of aging-related diseases, alternatives and complementary strategies to exercise programs are therefore essential.

Strategies that use exposure to hypoxia (low oxygen supply) by themselves or complementary to exercise programs have been suggested to benefit elderly people (Verges et al., 2015). Similar to exercise, hypoxia is a potent stimulus to induce molecular, metabolic and systemic adaptations that lead to an increased cellular and organism resilience, as long as this stimulus is mild enough to not cause permanent damage. Similar to regular exercise, repeated application of moderate hypoxia is associated with beneficial outcomes, including of antioxidant and anti-inflammatory defenses, cardiovascular and respiratory systems, etc. (J. Burtscher et al., 2022).

Recently, it has been proposed that the combination exercise and mild hypoxia allows a reduction of exercise intensity while maintaining normal training effects (Pramsohler et al., 2017). This approach reduces physical strain and thus may be particularly useful for load-compromised, older individuals (Girard et al., 2021).

While combined approaches are promising future avenues to increase the adherence of older people to exercise programs, exposure to hypoxia by itself may also promote healthy aging. Recent reviews support the notion that intermittent hypoxia-based interventions are efficient to maintain or even improve physical and cognitive performance, as well as metabolic and cardiovascular parameters (Tom Behrendt et al.) and highlight the potential for age-related diseases (J. Burtscher et al., 2022; Raberin et al., 2022).

However, the influence of hypoxia on age-related physiological changes, the different physiological mechanisms mediating these effects, the parameters of the protocols (e.g. severity and duration of hypoxia, frequency and patterns of applications) determining beneficial *versus* detrimental health outcomes and the basis for the high inter-individual variability in responses to hypoxia remain poorly understood. Better knowledge on all of these aspects is essential to unlock the full potential of hypoxia-based preventive and therapeutic interventions.

**FIGURE 1:**
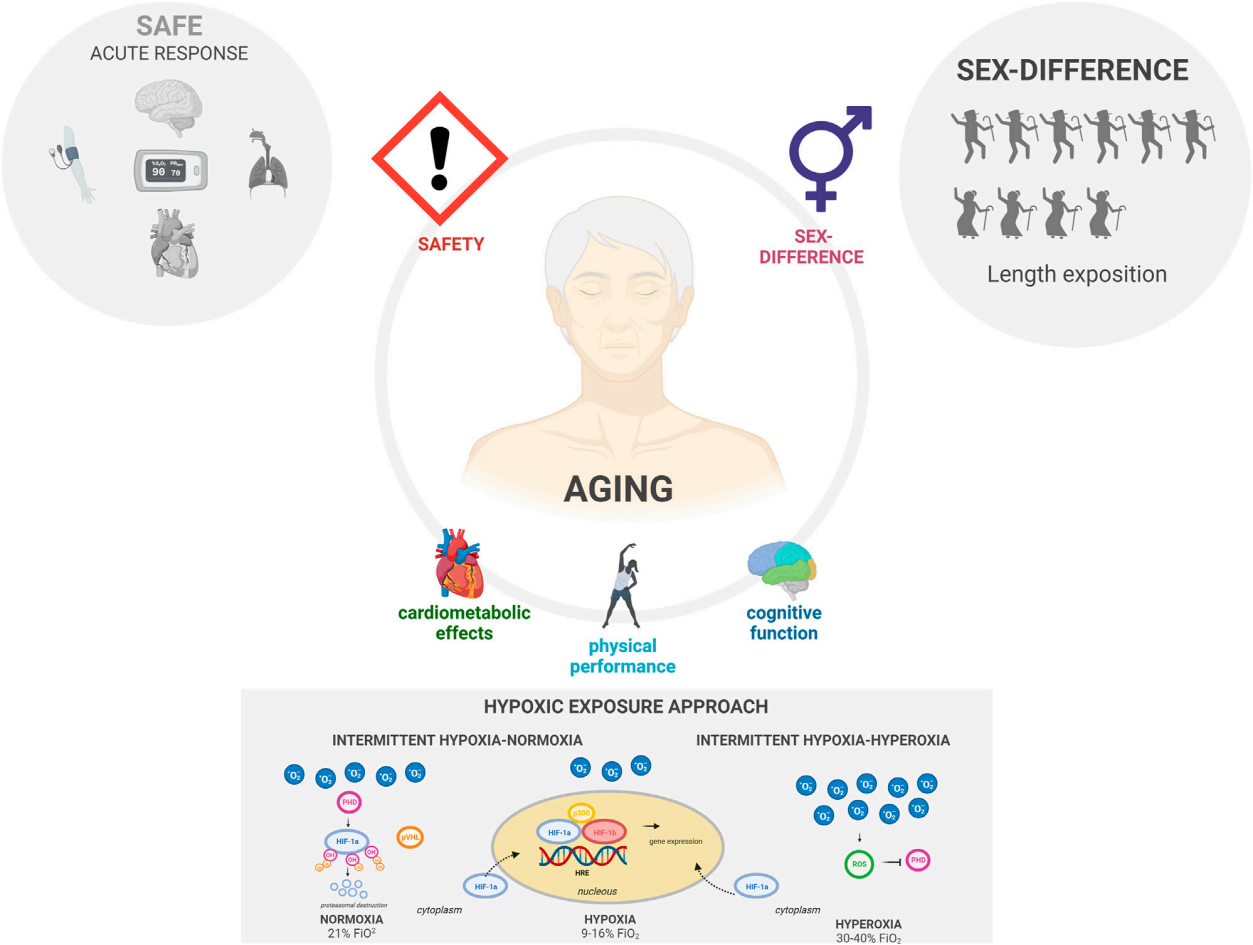
Hypoxia-Based interventions and ageing. Training in hypoxia has been shown as a safe therapy assessed through cardiac and pulmonary outcomes. Besides, substantially greater hypoxemia appears in elderly female compared with male, being need longer expositions in elderly male. Health benefits following exposure to hypoxia-based interventions in older people have been repeatedly reported (cardiometabolic effects, physical or cognitive performance are some examples). Different hypoxic exposure approach could have a different influence about hypoxia inducible factor (HIF): under normoxia (21% of fraction of inspired oxygen (FiO_2_)), proline hydroxylase (PHD) mediates the binding of the E3 ubiquitin ligase pVHL with HIF-a for proteasomal degradation in the presence of oxygen and iron; under hypoxic conditions (9%–16% FiO_2_), the hydroxylation of HIF-a is inhibited and it accumulates in the cytoplasm. Then, it translocates to the nucleus, where it dimerizes with the HIF-b subunit, binding to a highly conserved hypoxia response element (HRE) within promoters of hypoxia-responsive gene; under hyperoxia conditions (30%–40% FiO_2_), there is an exponential increase in reactive oxygen species (ROS), which inhibit PHD, allowing HIF to accumulate and translocate to the cell nucleus. Created with BioRender.

To shed light on these enigmas, this Research Topic aimed to collate the newest reports on hypoxia-based treatments combined or not with physical exercise in aging humans. We are proud to have received insightful results from some of the most influential groups of the research field. A total of five papers has been selected to be included into this Research Topic. The work presented in these papers arose from 35 different authors from eight countries and focuses on crucial topics, such as safety, protocol-optimization, acute responses to hypoxia, sex-differences, hypoxia and sleep quality, and the effects of hypoxic training on age-related health problems (Figure 1).

## 2 Acute responses, sex-differences, safety and protocols

Health benefits following exposure to intermittent hypoxia in older people have been repeatedly reported (M. Burtscher, 2004; Camacho-Cardenosa et al., 2019; Millet et al., 2016; Schega et al., 2016; Timon et al., 2021). Still, the impressive physiological outcomes (e.g., remarkable cardiovascular benefits; (Panza, Puri, Lin, Safwan Badr, et al., 2022)) reported in some studies are not yet well known in the general public and even in biomedical sciences. A certain degree of skepticism towards the application of hypoxia as a therapy is expected, especially since oxygen supplementation is a frequently used clinical intervention in patients with hypoxemia. In contrast, hypoxic therapies are not acute treatment strategies but instead induce acute hypoxic stress, which–in well-calibrated protocols–leads to beneficial adaptations that result in increased future resilience (J. Burtscher et al., 2022).

Knowing the physiological responses to acute hypoxia is crucial for the assessment of safety and efficiency of related procedures. In this Research Topic, (Panza et al.) provide important information on this aspect, by investigating the acute ventilatory and blood pressure response during and after 15 days of 12 bouts of 2 min of mild intermittent hypoxia (a representative protocol of intermittent hypoxia for therapeutic purposes) in patients with hypertension and obstructive sleep apnea. While ventilation remained unchanged, resting blood pressure was down-regulated after exposure to acute bouts of hypoxia, an important complementary finding to this authors’ group recently published study on the potential of intermittent hypoxia to reduce blood pressure in hypertensive males (Panza, Puri, Lin, Safwan Badr, et al., 2022).

While (Panza et al.) focused on males, (Zhao et al.) demonstrate that the consideration of sex is essential in the design of intermittent hypoxia interventions. They found substantially greater hypoxemia and chemoreflex sensitivity after 5 min of hypoxic breathing at 10% O_2_ in elderly females in comparison to men.

(Costa et al.) evaluated the effectiveness, tolerance and safety of exercising in hypoxia (inspired oxygen fraction of 13.5%) by monitoring peripheral oxyhemoglobin saturation, heart rate, rate of perceived exertion and blood lactate concentration in patients recovered from COVID-19. Based on these parameters, training in hypoxia was well-tolerated and safe.

(Furian et al.) highlight an important risk of prolonged hypoxia exposure, namely the potential aggravation of nocturnal hypoxemia, deoxygenations and impaired sleep quality (7-day sojourns at a high-altitude with 4–8 h/day at 2,900–5,050 m). These authors convincingly demonstrate that these detrimental effects can be mitigated by acclimatization and after recovery at 520 m.

In the last decades, exercise combined with alternating cycles of hypoxia and normoxia/hyperoxia has gained popularity as a promising non-pharmacological health-promoting approach for healthy, non-athletic populations. The use of interspersed, moderate hyperoxia may increase transcriptional programs in affected cells leading to even greater adaptive responses to hypoxia (Tom. Behrendt et al.).

Based on this assumption, in this Research Topic, (Behrendt et al.), asked, whether an intermittent hypoxic-hyperoxic exposure (IHHE) prior to aerobic cycling exercise 3 days per week for 6 weeks increases the effectiveness on cognitive and physical performance improvement in geriatric patients. In IHHE hypoxic air was administered for 1–5 min 4–8 times during one 30 min session, with each hypoxic episode being interspersed by a 1–3 min exposure to hyperoxia. Not only was IHHE well tolerated but in combination with the exercise program it also was more efficient in preserving/increasing cognitive and physical performance than exercise alone.

## 3 Conclusion

Well-calibrated hypoxia-based interventions alone or combined with physical exercise are promising strategies to slow down aging related decline. The articles in this Research Topic contribute to a better understanding of acute-effects, sex differences, potential risks and the design of safe and efficient protocols of intermittent hypoxia exposure. These and future investigations are crucial to optimize strategies utilizing the variation of oxygen supply to mitigate age-related decline and therefore render such approaches novel pillars in the prevention of morbidity in an aging population.
